# The Effect of Dietary Organic Copper and Zinc Trace Minerals on some Yield and Mineral Levels and Histological Structure of Testes

**DOI:** 10.1007/s12011-024-04114-7

**Published:** 2024-02-29

**Authors:** Vadullah Eren, Özay Güleş, Özdal Gökdal, Ülker Eren, Serap Ünübol Aypak

**Affiliations:** 1 Çine Vocational School, Aydın Adnan Menderes University, 09500 Çine, Aydın, Turkey; 2 Department of Histology and Embryology, Faculty of Veterinary Medicine, Aydın Adnan Menderes University, Aydın, Turkey; 3 Department of Biochemistry, Faculty of Veterinary Medicine, Aydın Adnan Menderes University, Aydın, Turkey

**Keywords:** Organic Copper and Zinc, Faeces, Fleece, Semen, Testes, Sheep

## Abstract

This study aims to investigate the effects of providing Cu and Zn minerals with an organic structure reduced by 25% compared to the recommended (NRC) inorganic value on parameters such as the age at which lambs achieve 50% sperm motility yield, some developmental parameters, testis histology, as well as serum, wool, and fecal mineral levels in lambs. The study involved 12 male lambs in the T1 group (organic minerals) and 11 in the T2 group (inorganic minerals) of the Kıvırcık breed. Lambs received minerals from mothers during the last month of fetal period and suckling, continuing individual feeding post-weaning. During individual feeding, T1 received 5.25 mg/kg DM copper-chelate and 15.0 mg/kg DM zinc-chelate, while T2 received 7 mg/kg DM copper-sulfate and 20 mg/kg DM zinc-sulfate. The mothers received identical mineral amounts in the last month of pregnancy and lactation. The ewes birthed offspring solely for the supply of experimental groups. Lambs, averaging about 18.5 kg, underwent bi-weekly electroejaculation, concluding the trial for those with 50% semen motility. Statistical analyses were carried out using the GLM method. No differences were observed between groups in the average age at which lambs achieve 50% sperm motility, live weight, scrotum, and testicular measurements at this age (*P* > 0.05). Histological analyses revealed no difference in tubule area between T1 and T2 groups (*P* > 0.05), but tubular epithelium height was greater in T1 (*P* < 0.01). End-of-trial serum copper, as well as weaning day and end-of-trial serum and fleece zinc mean values, did not differ between groups (*P* > 0.05). However, T1 had higher mean values for serum Cu on the weaning day (*P* < 0.01), fleece copper on the weaning day (*P* < 0.05) and at the end of the trial (*P* < 0.001). Additionally, the T1 group exhibited lower mean levels of fecal copper (*P* < 0.05) and fecal zinc (*P* < 0.001). In conclusion, despite organic copper and zinc levels being 25% lower in the examined parameters, comparable or improved results were achieved with inorganic copper and zinc.

## Introduction

Despite their low concentrations, trace minerals are essential for physiological functions. To avoid serious economic losses, the trace mineral requirement must be met [1, 2]. Copper and zinc are essential trace minerals for a healthy life [3]. These two elements play a very important role in the male and female reproductive systems [4, 5]. In addition to the central nervous system and the skeleton, copper plays an important role in pigment, hair, and fleece formation, as well as in various enzyme systems such as cytochrome oxidase [6, 7]. Although it is found in all cells, it is reported that copper accumulates more intensely in the fleece [2]. In its deficiency, anemia, diarrhea [8], inhibition of testosterone synthesis [9], pigment changes in hair and fleece [6, 7], disorders in glucose and lipid metabolism, and cardiovascular disorders occur [2, 7]. In animals with copper deficiency, seminiferous tubular development and spermatogenesis activity are reduced [10].

Zinc is an essential mineral that plays a vital role in many biological processes, such as enzyme activity, cell membrane stabilization, gene expression, and cell signaling [11, 12]. Zinc is an important component of the insulin hormone and is the building block of many metalloenzymes such as carbonic anhydrase, carboxypeptidase, alkaline phosphatase, and various dehydrogenases [2, 7]. Zinc plays an important role in ensuring sperm membrane integrity, increasing motility, and regulating sperm tail movements [5, 13]. It also plays a role in DNA replication, transcription, and protein synthesis, affecting cell division and differentiation [14]. Although it is found in all tissues, it is especially concentrated in bone, testis, prostate, skin, and fleece. However, it is noteworthy that it accumulates more intensely in fleece [2]. In zinc deficiency, disruptions in nucleic acid and protein synthesis [15], suppression of testosterone and follicle stimulating hormone (FSH) synthesis, as well as inhibition of spermatogenesis [16, 17] are observed by angiotensin enzyme inhibition and oxidative stress in the testis [18], depending on the increase in serum malondialdehyde (MDA) level as well as the decrease in serum superoxide dismutase (SOD) and glutathione peroxidase (GPx) levels [17].

It has also been determined that it increases germ cell apoptosis by causing an increase in TNF-α, Bax, and caspase 3 in the testis due to oxidative stress [17]. In addition, drying and cracking of the skin and epithelial tissue (parakeratosis), hair, and fleece shedding occur [2, 7]. Naderi et al. [19] determined that zinc application against valproic acid-induced testicular toxicity in rats increased the decreased testicular weight, sperm motility and number, testicular glutathione (GSH) level, and germinal cell layer thickness, and decreased the increased MDA level and sperm abnormality (%). Moreover, it minimized histopathological variations in the testis, such as vacuolization and degenerative changes in germinal cells.

Kheirandish et al. [13] found that zinc supplementation for 56 days prevented the shrinkage of seminiferous tubules and epithelial degeneration in mice exposed to copper intoxication. In addition, zinc and copper can prevent reactive oxygen compounds formed as a result of oxidative stress from causing cell damage in the organism by participating in the structure of SOD, an important antioxidant enzyme [20].

Trace minerals are used in inorganic structures and above normal NRC (National Research Council) values ​​in order to increase the yield in the rations of farm animals [21]. Inorganic salts such as oxides, sulfates, and carbonates are generally used in the diet. However, it has been reported that trace mineral compounds of organic origin are also being used, and due to the high absorption and bioavailability of organic compounds, optimum efficiency is obtained from animals in terms of growth, reproduction, and health [1, 22].

It is also stated that organic trace minerals are stored at higher concentrations in tissues and organs such as the blood, liver, bone, and kidney [23–25]. It has been reported that, as a result of more use of inorganic minerals, their excretion is intense and causes environmental pollution [26], while organic trace minerals are less excreted through feces because they are kept at a lower level in the rations [27–30].

The presented study is based on the hypothesis that similar results can be obtained when provided at a level lower than the level of inorganic minerals recommended by NRC [31] because their absorption and bioavailability in the organism increases when minerals are chelated (i.e., in organic form). Investigating potential outcomes in terms of possible economic benefits and reduction of environmental pollution is considered worthwhile. This study aims to investigate the effects of providing Cu and Zn minerals in an organically structured form, reduced by 25% from the recommended value for inorganic minerals [31], on parameters such as the age at which Kıvırcık lambs achieve 50% motility in sperm, live weight at that age, scrotum length, scrotum circumference, testis length, testis diameter, testis histology, as well as serum, wool, and fecal mineral levels.

## Materials and Methods

The current study was carried out with approval from the Aydin Adnan Menderes University Ethics Committee (Aydin, Turkey; no. 050.04/2010/46). The study included 12 male Kıvırcık lambs in the trial 1 (T1) group (receiving organic minerals) and 11 lambs in the trial 2 (T2) group (receiving inorganic minerals). These lambs were born from Kıvırcık ewes, who were fed individually with a ration containing organic or inorganic minerals and were not put out to pasture, during the last month of pregnancy and until the lambs were weaned.

Kıvırcık sheep is a multipurpose (meat, milk, and wool), long thin-tailed sheep breed of the Marmara and Aegean regions in Türkiye. A characteristic of Kıvırcık sheep is a white body coat. Kıvırcık sheep are known for the quality and tastiness of their lamb’s meat. In the big city markets in the Marmara region, there is an intense demand for meat of Kıvırcık lambs slaughtered immediately after the short suckling period which the meat in this period features a light pink color, thin muscle fiber, and a juicy taste [32].

The lambs that constituted the research material received minerals from their mothers in the last month of the fetal period and during the lactation period and continued to be fed individually after weaning until the end of the experiment. Organic minerals were given as copper chelate (5.25 mg/kg DM) and zinc chelate (15 mg/kg DM) to sheep and their lambs, and inorganic minerals were given as copper sulfate (7 mg/kg DM) and zinc sulfate (20 mg/kg DM) to sheep and their lambs [31]. Chelated forms of the minerals were given as organic metal salts of copper and zinc in the form of 2-hydroxy-4-methylthio butyrate. The organic mineral level was reduced by 25% from the inorganic mineral level. The weaned lambs were not allowed to graze during the individual feeding period.

The ration given to ewes and lambs is shown in Table 1. The intensive feed and vitamin-mineral mixture were weighed and given daily in a single meal, separately for each sheep and lamb. Wheat straw was given at two meals and after heavy feeding. Sheep were given 1500 g of dense feed (corn + soybean meal + vitamin-mineral mixture) and 1160 g of wheat straw per day. The lambs were initially fed 300 g of concentrated feed (corn + soybean meal + vitamin-mineral mix) and 200 g of wheat straw per day. The amounts of feed given increased due to the increase in weight of the lambs, and at the end of the experiment, 1500 g of dense feed and 1160 g of wheat straw were given per day. Water was given ad libitum.

**Table 1:** The composition of the ration given to ewe and lambs and the Cu and Zn values in wheat straw and compound feed

Feed material (%)	T1 group (Organic mineral)	T2 group (Inorganic mineral)
Wheat straw Sweet corn Soybean meal (44% CP) Vitamin-mineral mix*	43.53 30.85 23.22 2.40	43.53 30.85 23.22 2.40
Calculated, in DM		
ME, kcal/kg CP, g/kg	2112.78 162.3	2112.78 162.3
Wheat straw Cu (kg DM, ppm) Wheat straw Zn (kg DM, ppm) Mixed feed Cu (kg DM, ppm) Mixed feed Zn (kg DM, ppm)	7.58 8.54 7.39 17.13	7.58 8.54 7.39 17.13

*: in 1.0 kg of vitamin-mineral mix, 24 000 000 IU vitamin A, 4 800 000 IU vitamin D3, 48 000 mg vitamin E, 120 g salt, 320 g DCP, 80 g CaCO3, 960 mg manganese, 1920 mg iron, 24 mg of iodine, 4.80 mg of cobalt, 9.60 mg of selenium, 24 mg of molybdenum and 384 mg of magnesium. In addition, the inorganic mix contains 960 mg of zinc, and 336 mg of copper, while the organic mix contains 720 mg of zinc and 252 mg of copper (Sinerji Tarım Ürünleri San. Tic. Ltd.)

Lambs were individually weighed every 14 days using an electronic scale. Once the lambs reached an average live weight of about 18.5 kg, semen collection took place every 14 days through electroejaculation by using a rectal probe (Mark IV, Olivet, Ruakura, New Zealand). The trial ended for lambs exhibiting a 50% motility rate in the collected semen based on examination results. The scrotum length, scrotum circumference, length, and diameter of the right and left testes were measured with a metal caliper, and the lambs were sent to slaughter.

### Histological Analysis

Left testis tissue samples were taken from slaughtered lambs for histological examination and fixed in buffered formalin solution (NBF) for 24 h. Afterward, routine tissue follow-up was applied to testicular tissue samples, and they were blocked in paraffin. Six sections were taken from paraffin blocks, 6 μm thick, skipping 40 sections (with 240 μm intervals). Sections were taken, and a triple staining method that allows the tissue to be visible under the microscope was used for general histological examination and histometric measurements [33]. For histometric examination, 10 seminiferous tubular areas and epithelial heights were measured in each of the 6 sections taken for each animal [34]. Measurements were made interactively with the help of the image analysis program (Leica Q Win Standard) [35].

### Copper and Zinc Analysis

At the time of weaning and the end of the experiment, blood was collected from the jugular veins of the lambs. The separated serum was then preserved in a deep freezer at -20 °C. Blood samples were centrifuged at 3000 rpm for 5 min. The supernatants were separated to determine Cu and Zn levels. The supernatants were diluted 20 times in a 0.1 N HCl solution, and the copper and zinc levels in the blood serum were analyzed using spectrophotometry (Shimadzu Corp. UV-1601, Australia) with a kit (Randox, Cu: Cu2340 and Zn: Zn2341, Ardmore, United Kingdom). Fleece samples were obtained from the shoulder, rib, and thigh regions using shears, positioned close to the skin, during both the weaning process and at the end of the experiment. Stool samples were collected from the rectum with the assistance of fingers at the end of the trial. Until the mineral analysis of the samples, the feed and straw were stored in room conditions, fleece at + 4 ºC, and feces samples at -20 °C. The samples underwent processing through the wet burning method, and the copper and zinc levels were determined using ICP (Inductively Coupled Plasma Spectro-Optima 2100 DV ICP/OES, Perkin Elmer).

### Statistical Analysis

Normal distribution of the data of the body weight and testicular measurements was confirmed with the Shapiro-Wilk test and then compared by GLM in SAS (1998), considering group, time, and the interaction between treatment and time as the main effects. Time was included in the model as repeated data. Data are presented as the Least Squares Means ± SEM. The difference between groups in terms of the examined parameters was evaluated using the Student t-test for analysis of seminiferous tubule area and tubular epithelial height. Differences were considered as significant when *P* < 0.05 (*), *P* < 0.01 (**), or *P* < 0.001 (***) [36].

## Results

The mean values of age at achieving 50% sperm motility, live weight, scrotum length, scrotum circumference, testicular length, testicular diameter, seminiferous tubule area, and tubular epithelial height are provided in Table 2; while the mean values of Cu and Zn for serum, fleece, and feces are presented in Table 3.

**Table 2:** Least Squares Mean values of the age at which the groups have 50% motility in sperm production, and the corresponding live weight, scrotum length, scrotum circumference, testicular length, testicular diameter, seminiferous tubule area, and tubular epithelial height at this age

	T1 Group (Organic mineral) *n* = 12 (x ± s.e)	T2 Group (Inorganic mineral) *n* = 11 (x ± s.e.)	*P*
Age at achieving 50% sperm motility (day)	268.02 ± 13.13	264.90 ± 12.93	NS
Initial body weight	17.51 ± 0.95	19.44 ± 0.74	NS
Final body weight (kg)	42.99 ± 1.77	39.72 ± 1.74	NS
Scrotum length (cm)	15.21 ± 0.23	15.55 ± 0.22	NS
Scrotum circumference (cm)	29.16 ± 0.53	29.25 ± 0.52	NS
Testicular length (cm)	9.14 ± 0.19	9.40 ± 0.19	NS
Testicular diameter (cm)	5.21 ± 0.15	5.31 ± 0.15	NS
Seminiferous tubule area (µm^2^)	20273.45 ± 507.31	19224.20 ± 473.56	NS
Tubular epithelial height (µm)	50.33 ± 0.85	47.65 ± 0.50	**

X: mean, s.e.: standard error of mean (SEM), NS: not significant, **: *P* < 0.01

**Table 3:** Mean Cu and Zn values of serum and fleece at the weaning day and end of the trial, and mean Cu and Zn values in feces at the end of the trial in the groups

	T1 Group (Organic mineral) *n* = 12 (x ± s.e.)	T2 Group (Inorganic mineral) *n* = 11 (x ± s.e.)	*P*
**Serum Cu (µg dL-1)**			
Weaning Day	166.79 ± 6.04	133.24 ± 6.31	**
End of Trial	322.86 ± 23.70	276.27 ± 24.76	NS
**Serum Zn (µg dL-1)**			
Weaning Day	91.40 ± 5.28	91.70 ± 5.52	NS
End of Trial	185.88 ± 8.82	166.76 ± 9.21	NS
**Fleece Cu (ppm)**			
Weaning Day	8.67 ± 0.47	7.11 ± 0.49	*
End of Trial	11.04 ± 0.35	8.64 ± 0.37	***
**Fleece Zn (ppm)**			
Weaning Day	96.36 ± 4.58	84.25 ± 4.79	NS
End of Trial	105.15 ± 4.08	93.49 ± 4.26	NS
**Feces (ppm)**			
End of Trial Cu	21.24 ± 2.42	30.93 ± 2.52	*
End of Trial Zn	51.79 ± 5.04	81.15 ± 5.26	***

X: mean, s.e.: standard error of mean (SEM), NS: Not Significant, *: *P* < 0.05, **: *P* < 0.01, ***: *P* < 0.001

The average age at which the sperm of male lambs reached 50% motility was determined to be 268.02 ± 13.13 days in the T1 group and 264.90 ± 12.93 days in the T2 group (Table 2), with no difference observed between the group values (*P* > 0.05). No difference was observed between the groups in terms of initial and final body weight (kg), scrotum length (cm), scrotum circumference (cm), testicle length (cm), and testicular values measured on the days when the experiment ended (*P* > 0.05) (Table 2).

**Fig. 1:**
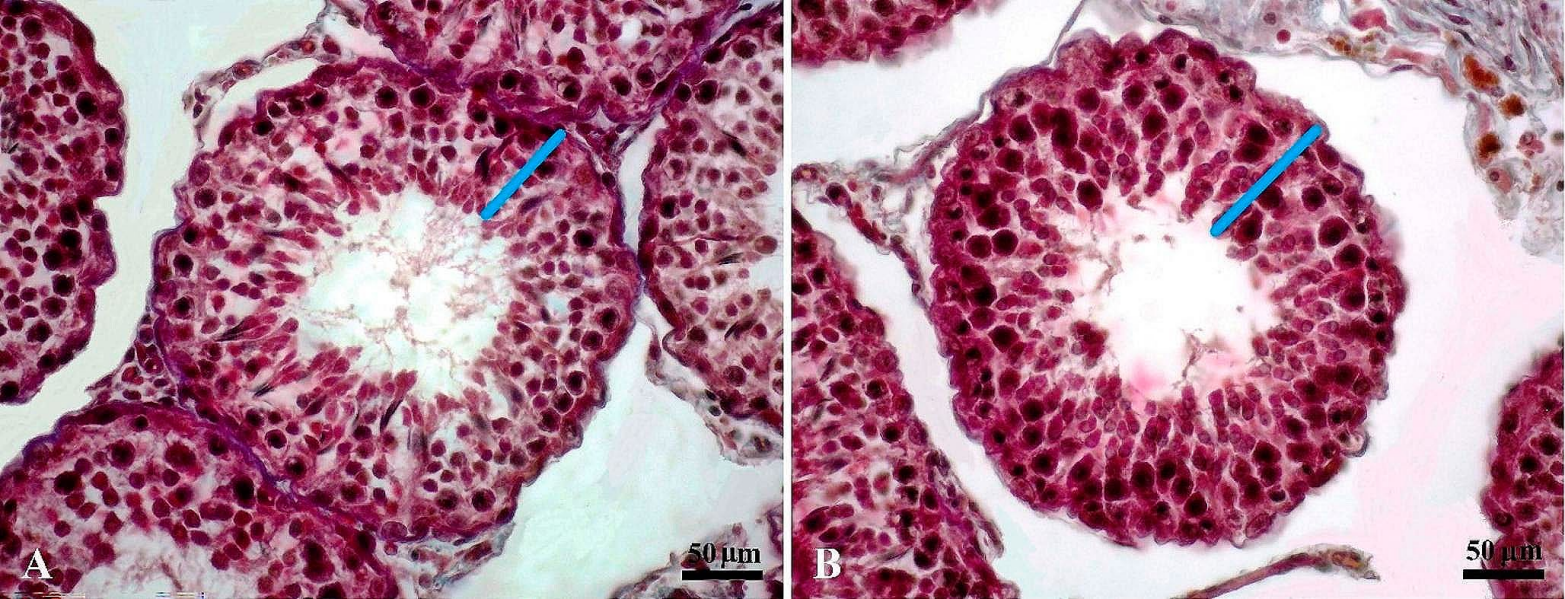
Seminiferous tubulus image of the T2-inorganic (**A**) and T1-organic (**B**) mineral groups. Crossman’s triple staining method

Microscopic examination of testicular tissues did not reveal any histological differences between the groups. In histometric analysis, the average area of seminiferous tubules in T1 was found to be similar to the values of the T2 group (*P* > 0.05). However, the height of the tubular epithelium increased in the T1 group (*P* < 0.01) (Fig. 1; Table 2).

The average values of serum (µg/dL), wool (ppm), and feces (ppm) for Cu and Zn recorded in the blood samples collected on the day of weaning and at the end of the trial are presented in Table 3. On the day of weaning, it was observed that the average values of serum copper (*P* < 0.01) and wool copper (*P* < 0.05) were higher in the T1 group (Table 3). At the end of the trial, it was observed that the measured average value of wool copper was higher (*P* < 0.001) in the group receiving organic Cu (*P* < 0.001). Trial end measurements revealed no differences among the average serum copper, serum zinc, and wool zinc values for the respective groups (*P* > 0.05). However, it was found that at the end of the trial, the average value of fecal copper (*P* < 0.05) and fecal zinc (*P* < 0.001) were lower in the T1 group.

## Discussion

In the presented study, the effects of using organic minerals instead of inorganic minerals, and even the effects of a 25% reduction in organic mineral usage, were examined. At the end of the research, initially, no significant difference was observed among groups in terms of the age at which lambs achieved 50% sperm motility (*P* > 0.05).

In a study carried out on Targhee rams, no difference was observed in sperm motility between groups receiving organic and inorganic zinc [37]. Also, in a study investigating the effect of trace mineral sources on bull semen quality, no difference was found between inorganic and organic mineral groups [38].

Additionally, at the end of the trial (on the day they provided 50% motile sperm), there was no significant difference observed in the average body weight values among the groups in the study (*P* > 0.05) (Table 2). Other researchers have reported similar findings. Shinde et al. [39] noted that the average daily weight gain of Malpura lambs, supplemented with Cu- and Zn-methionine, did not increase compared to the Cu- and Zn-sulfate group during the 115-day supplementation period. Similarly, Eren et al. [40] observed in Kıvırcık lambs, Esfiokhi et al. [41] in Zandi lambs, Wagner et al. [30] with calves, and Yost et al. [42] with heifers that the inclusion of organic or inorganic minerals in the diet did not result in a significant difference in live weight.

However, there are also studies indicating that calves weaned from milk and given zinc-methionine had a higher mean live weight compared to calves given zinc oxide [43, 44]. Garg et al. [45] recorded that the average daily gain of the lambs in the Zn-methionine group was significantly higher compared to the control and ZnSO4 groups (*P* < 0.05). In their study, Pal et al. [46] determined that in ewes, the gut absorption values, plasma, and liver tissue concentrations of Cu and Zn indicated better bioavailability for Cu- and Zn-methionine supplements compared to Cu- and Zn-sulfate. In this study, despite receiving organic minerals reduced by 25% compared to inorganic mineral levels in the T1 group, there was no significant difference (*P* > 0.05) between the groups mentioned above.

At the end of the trial, no significant differences (*P* > 0.05) were found among the groups in terms of scrotum length, scrotum circumference, testis length, and testis diameter. Studies have indicated that deficiencies in inorganic Zn and Cu hinder the development of the testes, leading to shrinkage and epithelial degeneration in the seminiferous tubule [10, 13]. In a study conducted on Sanjabi lambs [47], both organic (Zn methionine) and inorganic (zinc sulfate) zinc groups exhibited an increase in the height of the germinal epithelium in the testis compared to the control group; however, there was no significant difference between them (*P* > 0.05). However, in this study, it was observed that the histological appearance of the testis and seminiferous tubule areas in the group receiving organic Cu and Zn, with a 25% reduction in the recommended inorganic ratios, was similar to the T2 groups (*P* > 0.05). Moreover, it was determined that the height of the tubular epithelium was greater in the T1 group (*P* < 0.01), suggesting a positive impact on sperm production. Narasimhaiah et al. [48] have linked enhanced sperm production in goats to the administration of organic Cu and Zn, suggesting that this association is connected to the elevation of antioxidant enzymes and the reduction of oxidative stress. Indeed, Yao et al. [49] stated the widespread expression of Cu/ZnSOD and GPX3 in Leydig cells and seminiferous tubules, suggesting that a high level of Cu/ZnSOD and GPX3 may effectively address oxidative stress and contribute to spermatogenesis.

Copper absorption in ruminants is low, typically ranging from less than 1.0–10.0%, compared to nonruminants [2]. Gruzewska and Roussel [50] demonstrated that the copper present in the liver is associated with an organic compound, such as a protein, that is non-dialyzable. When copper is absorbed by the sheep, it is transferred to the liver and converted into hepatocuprein and then transformed into haemocuprein to be released into the bloodstream. Hepatocuprein or other Cu complexes in the liver may regulate the transfer of Cu from the dam to the fetus [51].

In the presented study, on the day of weaning, the average values of serum copper (*P* < 0.01) and wool copper (*P* < 0.05) were found to be higher in the group receiving organic Cu compared to the group receiving inorganic Cu (Table 3). This situation indicates that in this research, the trial group lambs received higher levels of organic Cu from their mothers during the last month of the fetal period and the suckling period, and also utilized it more efficiently compared to the group receiving inorganic Cu. Copper is present as part of the fat globule membrane materials in milk [52].

Indeed, in this study the wool copper levels at the end of the trial were significantly higher (*P* < 0.001) in the group receiving organic copper, providing evidence that lambs benefitted more efficiently from organic copper during their individual feeding period. The reason for this is likely due to the prevention of dissociation during passage through the digestive system and the enhanced biological availability of the mineral, possibly arising from the formation of chelates with organic ligands [53].

In chelation, metal ions form a secure bond with organic molecules (ligands), creating a protective ring structure that prevents the mineral element from engaging in undesired chemical reactions. The chelating ligand supplies at least two donor groups for metal binding – typically an amino group for a complex covalent bond and a carboxyl group for an ionic bond. The ligands need to be adequately spaced to allow the formation of a double heterocyclic ring compound. In this state, minerals can easily traverse the intestinal wall into the bloodstream, leading to increased metabolism of the mineral [54].

On the day of weaning, serum zinc values were not found to differ among the groups (*P* < 0.05). Also, at the end of the trial, although the mean values of serum copper and serum zinc were numerically higher in the group receiving organic minerals (Table 3), no difference was found between the groups (*P* > 0.05). Similar findings regarding serum copper and serum zinc levels have been reported in ram lambs and goats by Eren et al. [40, 55]. Whereas, Mallaki et al. [56] demonstrated that the level of zinc in the plasma of Zandi lambs increased in the Zn-peptide group compared to the Zn-sulphate group. Farghaly et al. [57] reported a significant increase (*P* < 0.05) in the serum zinc level in six-month-old lambs supplemented with Zn-methionine compared to the control and zinc sulfate groups. Also, Pal et al. [46] found that plasma Cu and Zn concentration was significantly higher (*P* < 0.06) in sheep consuming Cu-Meth + Zn-Meth compared to those supplemented with Cu-Sulf + Zn-Sulf sources.

The differences in results between our study and previous research might be due to how organic and inorganic zinc were evenly distributed among the study groups in those investigations. In our study, we intentionally took a different approach by administering organic zinc at a 25% lower rate compared to its inorganic counterpart. This intentional adjustment in treatment conditions could help explain the varied outcomes, underlining the importance of considering the method and dosage of zinc administration in future studies.

In the study, wool zinc values, both on the day of weaning and at the end of the trial, were numerically higher in the group receiving organic Zn, although this excess was not found to be significant (*P* > 0.05). This suggests that despite a 25% reduction, organic zinc is better retained. Lardy et al. [58] reported that organic zinc supplementation resulted in higher retention compared to inorganic zinc. This is attributed to the fact that organic zinc is transported intact from the intestinal lumen to mucosal cells, which may improve animal productivity by increasing tissue zinc supply [45].

At the end of the trial, the average values of Cu (*P* < 0.05) and Zn (*P* < 0.001) in the feces of the T1 group lambs were significantly lower compared to the T2 group (Table 3). This suggests that organic zinc and copper are retained and utilized in the body. Similarly, Pal et al. (2008) demonstrated a reduction (*P* < 0.01) in fecal excretion of copper and zinc when sourced from methionine-chelated compounds, indicating an increased utilization in the body. When viewed as a whole, serum, fleece, and fecal Cu and Zn values determined in T1 and T2 groups remain within the limits recorded for different sheep breeds [59, 60] and determined in studies conducted in our region [61, 62].

In conclusion, despite being provided at 25% lower levels in the lamb ration, organic copper and organic zinc were found to exhibit similar results to inorganic copper and inorganic zinc in terms of the examined parameters, and even demonstrated better performance in certain aspects. Additionally, these organic forms were observed to be excreted at lower levels in feces. The presented study suggests that providing copper and zinc in organic forms enhances their absorption and utilization in the body. The findings carry significance in both economic benefits and mitigating environmental pollution. Moving forward, it is believed that focusing on the transfer of organic minerals from mother to fetus during pregnancy or the effects of organic mineral intake on the mineral content in milk will contribute to the existing knowledge on this subject.

## Data Availability

No datasets were generated or analysed during the current study.

## References

[1] Spears JW (1996) Organic trace minerals in ruminant nutrition. Anim Feed Sci Tech 58:151–163. 10.1016/0377-8401(95)00881-0

[2] Underwood EJ, Suttle NF (1999) The mineral nutrition of livestock. CABI Publishing, UK

[3] Dieck HT, Doring F, Roth HP et al (2003) Changes in rat hepatic gene expression in response to zinc deficiency as assessed by DNA arrays. J Nutr 133:1004–1010. 10.1093/jn/133.4.1004 12672911 10.1093/jn/133.4.1004

[4] Herman S, Lipiński P, Ogórek M et al (2020) Molecular regulation of copper homeostasis in the male gonad during the process of spermatogenesis. Int J Mol Sci 21:9053. 10.3390/ijms21239053 33260507 10.3390/ijms21239053 PMC7730223

[5] Zhao J, Dong X, Hu X et al (2016) Zinc levels in seminal plasma and their correlation with male infertility: a systematic review and metaanalysis. Sci Rep 6:22386. 10.1038/srep22386 26932683 10.1038/srep22386 PMC4773819

[6] McDowell LR (1992) Minerals in animal and human nutrition. Academic Pres Inc, California

[7] Ergün A, Çolpan İ, Yıldız G et al (2020) Hayvan besleme ve beslenme hastalıkları. Pozitif Matbaacılık, Ankara

[8] Ueda M, Katsuse K, Kakumoto T et al (2023) Copper deficiency in Wilson’s disease with a normal zinc value. Intern Med 62:1073–1076. 10.2169/internalmedicine.9366-22 36047117 10.2169/internalmedicine.9366-22 PMC10125822

[9] Liu JY, Yang X, Sun XD et al (2016) Suppressive effects of copper sulfate accumulation on the spermatogenesis of rats. Biol Trace Elem Res 174:356–361. 10.1007/s12011-016-0710-7 27129317 10.1007/s12011-016-0710-7

[10] Van Niekerk FE, Van Niekerk CH (1989) The influence of experimentally induced copper deficiency on the fertility of rams. II. Macro- and microscopic changes in the testes. J S Afr Vet Assoc 60:32–35 2724285

[11] Swain PS, Rao SBN, Rajendran D et al (2016) Nano zinc, an alternative to conventional zinc as animal feed supplement: a review. Anim Nutr 2:134–141. 10.1016/j.aninu.2016.06.003 29767083 10.1016/j.aninu.2016.06.003 PMC5941028

[12] Uniyal S, Garg AK, Jadhav SE et al (2017) Comparative efficacy of zinc supplementation from different sources on nutrient digestibility, hemato-biochemistry and antioxidant activity in guinea pigs. Livest Sci 204:59–64. 10.1016/j.livsci.2017.08.009

[13] Kheirandish R, Askari N, Babaei H (2014) Zinc therapy improves deleterious effects of chronic copper administration on mice testes: histopathological evaluation. Andrologia 46:80–85. 10.1111/and.12047 23137167 10.1111/and.12047

[14] Frederickson CJ (1989) Neurobiology of zinc and zinc containing neurons. Int Rev Neurobiol 31:145–238. 10.1016/S0074-7742(08)60279-2 2689380 10.1016/s0074-7742(08)60279-2

[15] Croxford TP, McCormick NH, Kelleher SL (2011) Moderate zinc deficiency reduces testicular Zip6 and Zip10 abundance and impairs spermatogenesis in mice. J Nutr 141:359–365. 10.3945/jn.110.131318 21248196 10.3945/jn.110.131318 PMC3040901

[16] Joshi S, Nair N, Bedwal RS (2014) Dietary zinc deficiency effects dorso-lateral and ventral prostate of Wistar rats: histological, biochemical and trace element study. Biol Trace Elem Res 161:91–100. 10.1007/s12011-014-0053-1 25053558 10.1007/s12011-014-0053-1

[17] Omu AE, Al-Azemi MK, Al-Maghrebi M et al (2015) Molecular basis for the effects of zinc deficiency on spermatogenesis: an experimental study in the Sprague-Dawley rat model. Indian J Urol 31:57–64. 10.4103/0970-1591.139570 25624578 10.4103/0970-1591.139570 PMC4300574

[18] Chen Y, Yang J, Wang Y et al (2020) Zinc deficiency promotes testicular cell apoptosis in mice. Biol Trace Elem Res 195:142–149. 10.1007/s12011-019-01821-4 31309446 10.1007/s12011-019-01821-4

[19] Naderi M, Ahangar N, Badakhshan F et al (2021) Zinc and selenium supplement mitigated valproic acid-induced testis toxicity by modulating the oxidative redox balance in male rats. Anat Cell Biol 54:387–394. 10.5115/acb.20.280 34588319 10.5115/acb.20.280 PMC8493015

[20] Asadi S, Moradi MN, Khyripour N et al (2017) Resveratrol attenuates copper and zinc homeostasis and ameliorates oxidative stress in type 2 diabetic rats. Biol Trace Elem Res 177:132–138. 10.1007/s12011-016-0861-6 27744600 10.1007/s12011-016-0861-6

[21] İnal F, Coşkun B, Gulsen N (2001) The effects of withdrawal of vitamin and trace mineral supplements from layer diets on egg yield and trace mineral composition. Brit Poult Sci 42:77–80. 10.1080/713655024 11337972 10.1080/713655024

[22] Johnson AB, Socha M (1998) Judging trace mineral bioavailability. Feed Int 9:34–38

[23] Henry PR, Ammerman CB, Littell RC (1992) Relative bioavailability of manganese from a manganese-methionin complex and inorganic sources for ruminants. J Dairy Sci 75:3473–3478. 10.3168/jds.S0022-0302(92)78123-5 1474213 10.3168/jds.S0022-0302(92)78123-5

[24] Kincaid RL, Chew BP, Cronrath JD (1997) Zinc oxide and aminoacids as sources of dietary zinc for calves: effects on uptake and immunity. J Dairy Sci 80:1381–1388. 10.3168/jds.S0022-0302(97)76067-3 9241600 10.3168/jds.S0022-0302(97)76067-3

[25] Mezes M, Erdélyi M, Balogh K (2012) Deposition of organic trace metal complexes as feed additives in farm animals. Eur Chem Bull 1:410–423

[26] Leeson S (2003) A new look at trace minerals nutrition of poultry: Can we reduce environmental burden of poultry manure? In: Proceedings of the 19th Annual Symposium. Nottingham, United Kingdom, pp:125–131

[27] Nocek JE, Socha MT, Tomlinson DJ (2006) The effect of trace mineral fortification level and source on performance of dairy cattle. J Dairy Sci 89:2679–2693. 10.3168/jds.S0022-0302(06)72344-X 16772587 10.3168/jds.S0022-0302(06)72344-X

[28] Bao YM, Choct M, Iji PA et al (2008) Effect of organically complexed copper, iron, manganese and zinc on broiler performance, mineral excretion and accumulation in tissues. J Appl Poult Res 16:448–455. 10.1093/japr/16.3.448

[29] Nollet L, Huyghebaert G, Spring P (2008) Effect of different levels of dietary organic (Bioplex) trace minerals on live performance of broiler chickens by growth phases. J Appl Poult Res 17:109–115. 10.3382/japr.2007-00049

[30] Wagner JJ, Lacey JL, Engle TL (2008) The effect of organic trace minerals on feedyard performance and carcass merit in crossbred yearling steers. Prof Anim Sci 24:420–429

[31] National Research Council (1985) : Nutrient requirements of sheep. In: National Academiy of Sciences. Washington, USA, pp:43–78

[32] Gökdal Ö, Atay O, Eren V, Demircioğlu SK (2012) Fattening performance, carcass and meat quality characteristics of Kivircik male lambs. Trop Anim Health Prod 44:1491–1496. 10.1007/s11250-012-0093-5 22323106 10.1007/s11250-012-0093-5

[33] Crossman GA (1937) A modification of Mallory’s connective tissue stain with a discussion of the principles involved. Anat Rec 69:33–38. 10.1002/ar.1090690105

[34] Gules O, Dogan G, Ercins UH et al (2022) Effects of quercetin against doxorubicin-induced testicular toxicity in male rats. Biol Bull 49:203–213. 10.1134/S1062359022030086

[35] Gules O, Yildiz M, Naseer Z et al (2019) Effect of folic acid on testicular toxicity induced by bisphenol-A in male Wistar rats. Biotech Histochem 94:26–35. 10.1080/10520295.2018.1493222 30079777 10.1080/10520295.2018.1493222

[36] Statistical Analysis System [SAS] (1998) User’s guide. SAS Institute Inc, Cary, NC

[37] Page CM, Van Emon ML, Murphy TW et al (2020) Effects of zinc source and dietary concentration on serum zinc concentrations, growth performance, wool and reproductive characteristics in developing rams. Animal 14:520–528. 10.1017/S1751731119002180 31588886 10.1017/S1751731119002180

[38] Rowe MP, Powell JG, Kegley EB et al (2014) Effect of supplemental tracemineral source on bull semen quality. Prof Anim Sci 30:68–73. 10.15232/PAS.2018-01775

[39] Shinde AK, Sankhyan SK, Meena R et al (2013) Effect of feed supplementation with copper-and zinc salts on the growth, wool yield, nutrient utilization, blood constituents and mineral profile of Malpura lambs. Agric Sci Res J 3:284–291

[40] Eren V, Atay O, Gökdal Ö (2011) Organik bakır ve çinko’nun toklularda canlı ağırlık ile Bu minerallerin serum ve yapağıdaki düzeyleri üzerine etkisi. Kafkas Üniv Vet Fak Derg 17:95–99

[41] Esfiokhi SH, Sahlabadi MR, Khorrami B (2023) The study of chemical and nutritional characteristics of pea (Pisum sativum L.) pod silage and its effect on the performance of finishing Zandi lambs. J Anim Prod 25:375–388. 10.22059/jap.2023.363118.623753

[42] Yost GP, Arthington JD, McDowell LR et al (2002) Effect of copper source and level on the rate and extent of copper repletion in Holstein heifers. J Dairy Sci 85:3297–3303. 10.3168/jds.S0022-0302(02)74418-4 12512603 10.3168/jds.S0022-0302(02)74418-4

[43] Johnson BD, Hays VS, Gill DR et al (1988) Zinc methionine for newly recevied stocker cattle. Anim Sci Res Rep 125:111–116

[44] Spears JW, Hutcheson DP, Chirase NK (1991) Effects of zinc methionine and injectable copper pre-shipping on performance and health of stressed cattle. J Anim Sci 69:552

[45] Garg AK, Mudgal V, Dass RS (2008) Effect of organic zinc supplementation on growth, nutrient utilization and mineral profile in lambs. Anim Feed Sci Technol 144:82–96. 10.1016/j.anifeedsci.2007.10.003

[46] Pal DT, Gowda NKS, Prasad CS et al (2010) Effect of copper-and zinc-methionine supplementation on bioavailability, mineral status and tissue concentrations of copper and zinc in ewes. J Trace Elem Med Biol 24:89–94. 10.1016/j.jtemb.2009.11.007 20413065 10.1016/j.jtemb.2009.11.007

[47] Goodarzi N, Soroor MN, Rahimi-Feyli P et al (2018) Testicular stereology of lambs supplemented with organic and inorganic zinc. Bulgarian J Vet Med 21:301–312. 10.15547/bjvm.1070

[48] Narasimhaiah M, Arunachalam A, Sellappan S et al (2018) Organic zinc and copper supplementation on antioxidant protective mechanism and their correlation with sperm functional characteristics in goats. Reprod DomesT Anim 53:644–654. 10.1111/rda.13154 29450923 10.1111/rda.13154

[49] Yao T, Weng X, Liang W et al (2023) Differences of the anti-oxidative capability, GPX3, and Cu/ZnSOD expression in Hu sheep testis with different size at six-month-old. Anim Biotechnol 15:1–9. 10.1080/10495398.2023.2176317 10.1080/10495398.2023.2176317 PMC13353503 36794388

[50] Gruzewska Z, Roussel G (1937) Copper in liver proteins. Compte rendu des seances de la Societe de Biol 125:957–958

[51] Shearer GD, Innes JRM, McDougall EI (1940) Swayback studies in North Derbyshire: II. The relationship of the storage of copper and lead in the body tissues to the causation of Swayback. Vet J (1900) 96:309–322. 10.1016/S0372-5545(17)34904-0

[52] Murthy GK, Thomas JW (1974) Trace elements in milk. Crit Rev Environ Sci Technol 4:1–37

[53] Byrne L, Hynes MJ, Connolly CD et al (2021) Influence of the chelation process on the stability of organic trace mineral supplements used in animal nutrition. Animals 11:1730. 10.3390/ani11061730 34200569 10.3390/ani11061730 PMC8227544

[54] Roy RK, Misger FA (2008) Chelated minerals in livestock nutrition: a review. Environ Ecol 26:665

[55] Eren V, Gökdal O, Akşit H et al (2013) The effects of additional organic copper and organic zinc trace minerals on accumulation and elimination levels in female kids. Ankara Üniv Vet Fak Derg 60:89–92. 10.1501/Vetfak_0000002559

[56] Mallaki M, Norouzian MA, Khadem AA (2015) Effect of organic zinc supplementation on growth, nutrient utilization, and plasma zinc status in lambs. Turkish J Vet Anim Sci 39:75–80. 10.3906/vet-1405-79

[57] Farghaly MM, Mousa SM, El-Hafez A et al (2017) The effect of zinc supplementation on performance of growing lambs. Egypt J Nutr Feeds 20:59–68. 10.21608/EJNF.2017.103924

[58] Lardy G, Kerley MS, Patterson JA (1992) Retention of chelated metal proteinates by lambs. J Anim Sci 70:314

[59] Pope AL (1971) A review of recent mineral research with sheep. J Anim Sci 33:1332–1343 4947562 10.2527/jas1971.3361332x

[60] Teixeira IAMDA, Resende KTD, Silva AMDA et al (2013) Mineral requirements for growth of wool and hair lambs. R Bras Zootec 42:347–353. 10.1590/S1516-35982013000500007

[61] Tiftik AM, Doğanay ÇS (1997) İzmir bölgesi koyunlarında Kan Serumu bakır (Cu), demir (Fe), total demir bağlama kapasitesi (tdbk) ve çinko (zn) düzeylerinin araştırılması. Vet Bil Derg 13:147–156

[62] Kargin F, Seyrek K, Bildik A et al (2004) Determination of the levels of zinc, copper, calcium, phosphorus and magnesium of Chios ewes in the Aydın region. Turkish J Vet Anim Sci 28:609–612

